# Impact of Axial Length on Visual Outcomes and OCT Findings in Epiretinal Membrane: A Retrospective Study

**DOI:** 10.3390/jcm15114360

**Published:** 2026-06-04

**Authors:** Reo Sueno, Noriko Kubota, Yosai Mori, Kazunori Miyata, Yuji Nakano, Hitoshi Goto, Tomoyuki Kunishige, Fumiki Okamoto

**Affiliations:** 1 Department of Ophthalmology, Nippon Medical School, 1-1-5 Sendagi, Bunkyo-ku, Tokyo 113-8603, Japan; reo-sueno@nms.ac.jp (R.S.); f-okamoto@nms.ac.jp (F.O.); 2 Miyata Eye Hospital, Miyazaki 885-0051, Japan; 3 Department of Ophthalmology, Nippon Medical School Tama Nagayama, Tokyo 206-8512, Japan

**Keywords:** epiretinal membrane, axial length, high myopia, optical coherence tomography, vitrectomy, cystoid lesions, visual prognosis

## Abstract

**Background:** Axial elongation may influence the structural characteristics and surgical prognosis of epiretinal membrane (ERM), but its impact across different axial length (AL) categories remains unclear. **Methods:** We retrospectively analyzed 557 eyes from 515 patients with ERM who underwent vitrectomy and were followed for at least 6 months. Eyes were stratified by AL into normal to mild myopia (NM; <26 mm), high myopia (HM; ≥26 to <28 mm), and extreme high myopia (EHM; ≥28 mm). Clinical variables included age, AL, and pre- and postoperative best-corrected visual acuity (BCVA). OCT parameters assessed included inner and outer retinal cystoid lesions, epiretinal proliferation (EP), and ellipsoid zone (EZ) disruption. **Results:** Postoperative BCVA significantly improved in the NM and HM groups (*p* < 0.001) but not in the EHM group (*p* = 0.091). Although preoperative BCVA was comparable among groups, postoperative BCVA was significantly better in the HM group (−0.02 ± 0.17 logMAR) compared with the NM (0.03 ± 0.20 logMAR) and EHM (0.21 ± 0.43 logMAR) groups (*p* < 0.05). Outer retinal cystoid lesions were significantly more frequent in the EHM group (*p* < 0.05), whereas the prevalence of EZ disruption, inner retinal cystoid lesions, and EP did not differ significantly among groups. **Conclusions:** Eyes with ERM and extreme high myopia exhibited limited postoperative visual improvement and a higher prevalence of outer retinal cystoid lesions, suggesting that axial elongation may adversely affect surgical prognosis.

## 1. Introduction

Epiretinal membrane (ERM) is characterized by the proliferation of fibrocellular tissue on the internal limiting membrane (ILM), leading to retinal wrinkling, macular traction, and subsequent visual disturbances such as decreased visual acuity and metamorphopsia [1,2,3]. Recent advances in optical coherence tomography (OCT) have provided detailed insights into the microstructural alterations associated with ERM, and comprehensive reviews have emphasized the importance of OCT-based biomarkers in predicting surgical outcomes [4]. ERM is a common vitreoretinal disorder, with a prevalence ranging from 2.2% to 28.9% in population-based studies [5,6,7,8,9]. High myopia has emerged as a major global public health concern, with increasing prevalence worldwide and a growing burden of myopia-related macular complications [10]. Axial elongation in highly myopic eyes induces structural changes in the vitreoretinal interface, including early posterior vitreous detachment (PVD), posterior staphyloma formation, and chorioretinal atrophy, all of which may influence the development and progression of ERM as well as surgical outcomes [11,12]. Previous studies have shown that longer axial length (AL) is associated with pathologic myopia and visual impairment [6,13,14]. During ERM surgery, ILM peeling is routinely performed; however, retinal thinning and posterior staphyloma in highly myopic eyes may increase technical complexity and potentially affect postoperative visual recovery. Although vitrectomy for ERM is widely performed, the association between axial length and postoperative visual outcomes remains incompletely understood. Most previous studies have primarily evaluated outcomes based on central retinal thickness measurements, while relatively few have investigated detailed microstructural changes in relation to axial length stratification [15,16,17,18]. Moreover, recent studies have highlighted that specific OCT-based microstructural parameters, such as ectopic inner foveal layers (EIFL) and tractional configurations, are closely associated with visual prognosis in ERM-related disorders [19]. However, it remains unclear whether the distribution of these microstructural features differs across axial length categories and how such differences relate to postoperative visual outcomes. Therefore, the present study aimed to investigate OCT-based microstructural characteristics and postoperative visual outcomes in patients with ERM by stratifying eyes according to axial length into normal to mild myopia, high myopia, and extreme high myopia groups. Through this stratified analysis, we sought to clarify the association between axial length and both structural changes and functional outcomes in ERM.

## 2. Methods

### 2.1. Ethics Approval

This retrospective study was conducted with approval from the Ethics Committee of Nippon Medical School (approval number: M-2023-161) [20]. The study adhered to the ethical standards of our institution. Written informed consent was obtained from all participants for the use of their clinical data for research purposes. All procedures were performed in accordance with the principles outlined in the Declaration of Helsinki.

### 2.2. Patient Characteristics and OCT Assessment

We conducted a retrospective review of consecutive patients diagnosed with ERM who underwent vitrectomy between April 2020 and December 2023. All patients were followed for a minimum of 6 months after surgery. Exclusion criteria included prior vitreoretinal surgery, secondary ERM due to retinal vascular disease, uveitis, trauma, or other ophthalmic disorders except for mild cataract, as well as eyes with moderate to severe cataract, corneal abnormalities, or systemic conditions known to affect visual acuity. Clinical details collected from the medical records included patients’ age, gender, and best-corrected visual acuity (BCVA). ERM was diagnosed using spectral-domain OCT (Spectralis version 1.8.6.0, Heidelberg Engineering GmbH, Heidelberg, Germany). Eyes were categorized according to AL into normal to mild myopia (NM; <26 mm), high myopia (HM; ≥26 to <28 mm), and extreme high myopia (EHM; ≥28 mm). OCT-based microstructural parameters assessed included the presence of inner and outer retinal cystoid lesions, epiretinal proliferation (EP) and disruption of ellipsoid zone (EZ). Outer and inner retinal cystoid lesions were defined as hyporeflective spaces within the Henle’s fiber layer or outer plexiform layer, and within the inner nuclear layer, respectively, as visualized by spectral-domain OCT. EZ disruption was defined as the presence of discontinuity or irregularity in the ellipsoid zone within a 1 mm diameter region centered on the fovea. BCVA was assessed both before and after surgery using a standard Japanese decimal visual acuity chart at a testing distance of 5 m. For statistical evaluation, decimal visual acuity values were converted into logarithm of the minimal angle of resolution (logMAR) units. AL was determined using ultrasonography (AL-4000; Tomey Corporation, Nagoya, Japan).

### 2.3. Surgical Methods

All procedures were carried out under local anesthesia using a 25-gauge pars plana vitrectomy system by two experienced vitreoretinal surgeons (F.O. and Y.M.). Following confirmation of complete posterior vitreous detachment, vitrectomy was completed, after which 0.1–0.2 mL of 0.025% brilliant blue G dye was carefully applied to the macular surface and subsequently removed by irrigation. Following ERM removal, the macular area was stained with brilliant blue G to facilitate visualization of the internal limiting membrane (ILM), which was subsequently peeled. In eyes with epiretinal proliferation (EP), the EP was embedded along with the ILM into the foveal defect, while the surrounding ILM was detached. When required, phacoemulsification and intraocular lens implantation were performed prior to vitrectomy.

### 2.4. Statistical Methods

Mean values and standard deviations were computed for age, AL, and BCVA before and after surgery. Differences in pre- and postoperative BCVA were assessed using the Wilcoxon signed-rank test, and intergroup comparisons among axial length categories were performed using the Kruskal–Wallis test. Associations between categorical variables, such as the distribution of retinal findings, were analyzed using Fisher’s exact test. The correlation between axial length and change in BCVA was evaluated using Spearman’s rank correlation coefficient. Because the distribution of BCVA change was not normal, non-parametric methods were used. Categorical variables, including the distribution of AL groups and the presence of inner and outer retinal cystoid lesions, EP, and EZ disruption, were compared using Fisher’s exact test. A *p*-value < 0.05 was considered statistically significant. All statistical analyses were performed using SPSS Statistics (version 29.0, IBM Corp., Armonk, NY, USA).

## 3. Results

A total of 557 eyes from 515 patients (264 males and 251 females; mean age, 71.4 ± 8.2 years [mean ± SD]) were included. The NM, HM, and EHM groups consisted of 496 (89.0%), 51 (9.2%), and 10 (1.8%) eyes, respectively. Table 1 summarizes demographic data, preoperative BCVA and OCT parameters. Patients in the NM group were significantly older than those in the HM and EHM groups. Preoperative BCVA did not differ significantly among the three groups. Preoperative outer retinal cystoid lesions were significantly more frequent in the EHM group compared with the other groups (*p* < 0.05). Preoperative presence of inner retinal cystoid lesions was not observed in the EHM group, while preoperative EZ disruption tended to be more common in the EHM group. Preoperative presence of EP was observed only in the NM group. No significant intergroup differences were found for inner retinal cystoid lesions, EZ disruption, or EP. Combined phacoemulsification and vitrectomy were performed in 99.2% (487/491) of eyes in the NM group, 98.0% (50/51) in the HM group, and 100% (9/9) in the EHM group, with no significant intergroup difference (Fisher’s exact test, *p* = 0.439).

**Figure 1:**
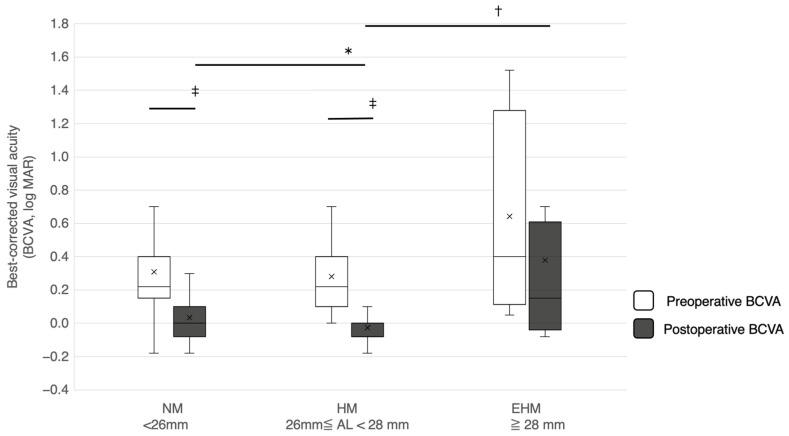
Best-corrected visual acuity (BCVA) before and after surgery in patients with epiretinal membrane (ERM) classified into three groups: normal to mild myopia (NM), high myopia (HM), and extreme high myopia (EHM). Postoperative BCVA significantly improved in the NM and HM groups, while no significant improvement was noted in the EHM group. No significant difference was found among the three groups preoperatively. Postoperatively, BCVA in the HM group was significantly better than in both the NM and EHM groups. In each box, the horizontal line indicates the median, and the “×” symbol represents the mean value. * *p* < 0.05, † *p* < 0.01, ‡ *p* < 0.001, Wilcoxon signed rank test, Kruskal–Wallis test.

Figure 1 shows pre- and postoperative BCVA in all three groups. The mean improvement in BCVA was 0.28 logMAR in the NM group, 0.30 logMAR in the HM group, and 0.43 logMAR in the EHM group, corresponding to approximately 2.8, 3.0, and 4.3 lines of visual acuity improvement, respectively.

Postoperative BCVA was significantly better in the HM group compared with both the NM (*p* < 0.05) and EHM (*p* < 0.005) groups, whereas no significant difference was observed between the NM and EHM groups. Significant improvement in postoperative BCVA following surgery was observed only in the NM (*p* < 0.001) and HM (*p* < 0.001) groups, whereas no significant difference was seen in the EHM group (*p* = 0.091). Postoperatively, BCVA in the HM group (−0.02 ± 0.17) was significantly better than that in both the NM (0.03 ± 0.20) and EHM (0.21 ± 0.43) groups (*p* < 0.05), whereas no significant difference was found between the NM and EHM groups.

**Figure 2:**
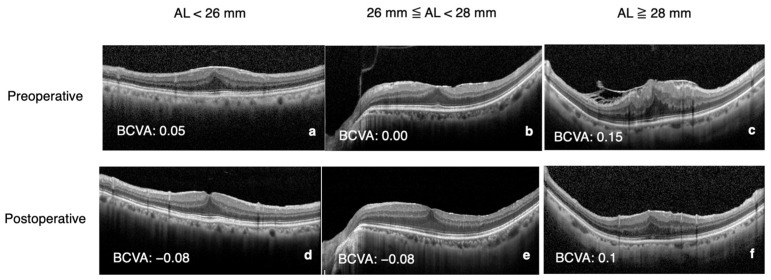
Representative preoperative (**a**–**c**) and postoperative (**d**–**f**) OCT images of eyes with epiretinal membrane (ERM) in the normal to mild myopia (NM), high myopia (HM), and extreme high myopia (EHM) groups.

Figure 2 presents representative pre- and postoperative OCT images. In the NM example (AL 23.07 mm), BCVA improved from 0.05 to −0.08 logMAR. In the HM case (AL 26.67 mm), BCVA improved from 0.00 to −0.08. In the EHM case (AL 28.35 mm), BCVA changed from 0.15 to 0.10. These images illustrate the structural differences across AL groups and highlight the limited functional recovery observed in EHM eyes.

**Figure 3:**
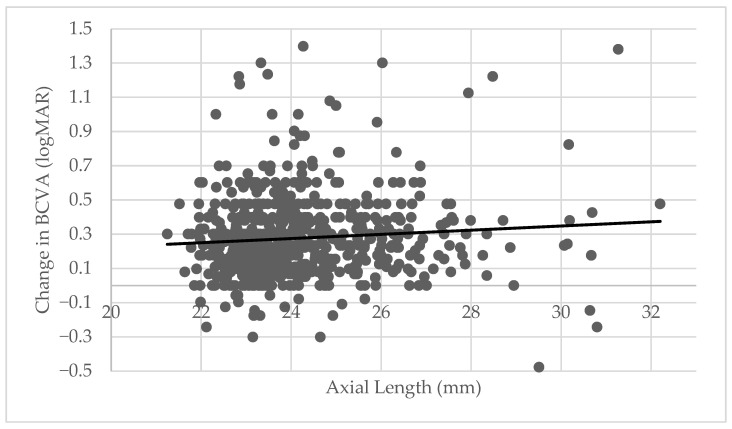
Scatter plot showing the relationship between axial length and change in best-corrected visual acuity (logMAR) after surgery. Each point represents an individual eye. A very weak but statistically significant correlation was observed (Spearman’s ρ = 0.087, *p* = 0.027).

Figure 3 shows the relationship between axial length and the change in BCVA. A weak but statistically significant positive correlation was observed between axial length and the change in BCVA (Spearman’s ρ = 0.087, *p* = 0.027), indicating that eyes with longer axial length tended to show slightly greater visual improvement.

## 4. Discussion

In this study, we evaluated OCT-based microstructural features and visual outcomes of eyes with ERM across axial length categories, demonstrating distinct clinical and morphological profiles among the NM, HM, and EHM groups. These findings suggest that axial length is associated with differences in disease phenotype and postoperative prognosis. However, the scatter plot analysis revealed no strong linear correlation between axial length and the change in BCVA, despite a statistically significant but very weak association (Spearman’s ρ = 0.087, *p* = 0.027). These findings suggest that postoperative visual improvement is influenced by multiple factors beyond axial length alone.

Consistent with previous reports showing that ERM arises at a younger age in highly myopic eyes [15], patients in the HM and EHM groups in our study were significantly younger than those in the NM group. This may be attributed to the earlier onset of posterior vitreous detachment (PVD) in eyes with longer axial length, which reduces vitreoretinal adhesion and may accelerate the onset of ERM [10].

Postoperative BCVA was significantly better in the HM group than in the NM and EHM groups in our results. This degree of improvement corresponds to approximately three lines of visual acuity gain, indicating a clinically meaningful functional benefit following surgery. The relatively favorable prognosis in HM may be attributable to reduced tractional damage due to early PVD and relative preservation of macular microstructure [18]. In contrast, the EHM group exhibited a markedly higher frequency of outer retinal cystoid lesions, which appeared to be associated with poorer visual outcomes. Structural alterations in the choroid, such as undulations of Bruch’s membrane, have been reported in highly myopic eyes related to longer axial length [21]. Outer retinal cystoid lesions were consistently located in the outer nuclear layer and may have resulted from posterior staphyloma–related traction acting predominantly on the outer retina in this study. Previous studies have demonstrated that axial length is a key determinant of myopic maculopathy, with pathological changes becoming more prevalent in eyes with axial length exceeding 28 mm [22]. Furthermore, posterior staphyloma, a hallmark of advanced myopic degeneration, has been shown to be associated with longer axial length and worse visual acuity [22]. These findings support the clinical relevance of axial length stratification in evaluating disease severity and prognosis [22]. Posterior staphyloma was observed in 7 of 9 EHM cases, and all 5 eyes with outer retinal cystoid lesions also had posterior staphyloma, supporting the hypothesis that longer axial length and posterior staphyloma may increase tractional stress on the outer retina and promote cyst formation. However, posterior staphyloma was not systematically evaluated in all cases in this study, which limits the ability to establish a definitive relationship between staphyloma and outer retinal cyst formation. Therefore, the observed association should be interpreted with caution, and further prospective studies with standardized assessment are warranted.

Prior investigations have indicated that cystoid changes in ERM and highly myopic eyes are closely associated with tractional stress on the retina rather than simple intraretinal fluid accumulation. Shiode et al. reported that outer retinal cystoid lesions located in Henle’s fiber layer and the outer plexiform layer typically resolved after ERM surgery, whereas inner retinal cystoid lesions within the inner nuclear layer tended to persist, reflecting irreversible Müller cell damage [22]. Their findings suggest that cyst location correlates with the degree of tractional stress and postoperative prognosis. Although outer retinal cystoid lesions in idiopathic ERM are generally reversible, the outer cystoid lesions observed in our EHM group may represent a distinct pathological mechanism. In eyes with extreme high myopia, chronic traction related to posterior staphyloma and structural alterations associated with longer axial length may lead to persistent cystoid alterations and photoreceptor impairment, resulting in poorer postoperative visual outcomes despite surgical release of traction. Recent studies have also highlighted that microstructural tractional changes at the fovea influence visual prognosis after ERM surgery. Mafi et al. demonstrated that the presence and severity of EIFL were strongly associated with postoperative visual function and served as a key biomarker of traction-induced inner retinal remodeling [23]. Notably, in our cohort, there were no significant differences in the prevalence of EIFL among the three axial-length groups, suggesting that EIFL alone does not account for the inferior visual outcomes observed in the EHM group. Instead, our results indicate that outer retinal involvement (particularly outer cyst formation associated with posterior staphyloma) may play a more critical role in determining postoperative visual outcomes in eyes with extreme axial elongation. Additional evidence from prior studies further supports the role of tractional stress in the formation of cystoid changes in highly myopic eyes. Kamal-Salah et al. demonstrated that paravascular retinal cystoid lesions and retinoschisis in highly myopic eyes were associated with tractional structures such as ERM and posterior staphyloma [24]. Similarly, Kubota et al. showed that outer retinal cystoid lesions were markedly more frequent in eyes with ERM-associated foveoschisis than in typical ERM, suggesting that mechanical traction on the outer retina plays a major role in cyst formation in eyes with elongated axial length [25].

This study has several limitations. First, the number of eyes in the EHM group was relatively small, limiting the statistical power of subgroup analyses. Second, axial length was measured using ultrasonography rather than an optical biometry device such as swept-source OCT. Although optical biometry is currently preferred for its higher precision, ultrasonographic measurement has been widely used and validated in clinical practice. Third, posterior staphyloma was not assessed in all cases, restricting evaluation of its interaction with cyst formation. Finally, the majority of surgeries included combined vitrectomy and cataract surgery, which may influence postoperative visual outcomes. Although strict exclusion criteria were applied, residual confounding due to unmeasured factors cannot be completely excluded. However, the proportion of combined cataract surgery did not differ significantly among the axial length groups.

Despite these limitations, our findings provide meaningful clinical insights. Eyes with ERM and extreme high myopia demonstrated a higher risk of outer retinal cyst formation and poorer postoperative visual improvement. These results underscore the importance of axial length as a stratification factor in preoperative risk assessment and patient counseling. Furthermore, the presence of outer retinal cystoid lesions may serve as a potential prognostic biomarker for surgical outcomes in ERM, meriting further investigation in larger, prospective studies.

## 5. Conclusions

Eyes with ERM and extreme high myopia exhibited poorer postoperative visual outcomes and a higher frequency of outer retinal cystoid lesions compared with eyes in the NM and HM groups. Axial length stratification revealed distinct structural and functional characteristics among ERM eyes, suggesting that extreme axial elongation contributes to outer retinal vulnerability and limited visual recovery after surgery. Preoperative assessment of axial length and outer retinal microstructure may improve surgical risk stratification and patient counseling in ERM management.

## Figures and Tables

**Table 1:** Clinical characteristics and preoperative optical coherence tomography (OCT) parameters of patients with epiretinal membrane classified by axial length.

Axial Length (mm)	Normal-Mild Myopia	High Myopia	Extreme High Myopia	*p*-Value
	<26	≥26 AL < 28	≥28	
Number of eyes, *n* (%)	496 (89.0%)	51 (9.2%)	10 (1.8%)	
Age (years ± SD)	72.3 ± 7.7	63.8 ± 8.6	64.8 ± 9.5	<0.001 ‡
Preoperative BCVA	0.31 ± 0.27	0.28 ± 0.31	0.64 ± 0.60	0.255
Postoperative BCVA	0.03 ± 0.20	−0.02 ± 0.17	0.21 ± 0.43	<0.01 †
Preoperative OCT parameters				
Outer retinal cystoid lesions (%)	67 (13.5%)	5 (9.8%)	4 (44.4%)	<0.05 *
Inner retinal cystoid lesions (%)	51 (10.2%)	4 (7.8%)	0 (0.0%)	0.517
EZ disruption (%)	82 (16.6%)	10 (19.6%)	3 (33.3%)	0.356
EP (%)	13 (2.6%)	0 (0.0%)	0 (0.0%)	0.448

BCVA = best-corrected visual acuity, OCT = optical coherence tomography, SD = standard deviation, EP = epiretinal proliferation, EZ = ellipsoid zone. Values are presented as the mean ± standard deviation. Significantly different from the other groups (* *p* < 0.05, † *p* < 0.01, ‡ *p* < 0.001, Kruskal–Wallis test with post hoc pairwise comparisons, Bonferroni correction).

## Data Availability

The data presented in this study are available from the corresponding author upon reasonable request.
